# Modeling regulatory cascades using Artificial Neural Networks: the case of transcriptional regulatory networks shaped during the yeast stress response

**DOI:** 10.3389/fgene.2013.00110

**Published:** 2013-06-20

**Authors:** Maria E. Manioudaki, Panayiota Poirazi

**Affiliations:** ^1^Institute of Molecular Biology and Biotechnology, Foundation for Research and Technology-HellasHeraklion, Crete, Greece; ^2^Department of Chemistry, University of CreteHeraklion, Crete, Greece

**Keywords:** Artificial Neural Networks, transcriptional regulatory networks, yeast stress response, three layers regulatory cascades, asynchronous regulation, heterodimers

## Abstract

Over the last decade, numerous computational methods have been developed in order to infer and model biological networks. Transcriptional networks in particular have attracted significant attention due to their critical role in cell survival. The majority of network inference methods use genome-wide experimental data to search for modules of genes with coherent expression profiles and common regulators, often ignoring the multi-layer structure of transcriptional cascades. Modeling methodologies on the other hand assume a given network structure and vary significantly in their algorithmic approach, ranging from over-simplified representations (e.g., Boolean networks) to detailed -but computationally expensive-network simulations (e.g., with differential equations). In this work we use Artificial Neural Networks (ANNs) to model transcriptional regulatory cascades that emerge during the stress response in *Saccharomyces cerevisiae* and extend in three layers. We confine the structure of the ANNs to match the structure of the biological networks as determined by gene expression, DNA-protein interaction and experimental evidence provided in publicly available databases. Trained ANNs are able to predict the expression profile of 11 target genes across multiple experimental conditions with a correlation coefficient >0.7. When time-dependent interactions between upstream transcription factors (TFs) and their indirect targets are also included in the ANNs, accurate predictions are achieved for 30/34 target genes. Moreover, heterodimer formation is taken into account. We show that ANNs can be used to (1) accurately predict the expression of downstream genes in a 3-layer transcriptional cascade based on the expression of their indirect regulators and (2) infer the condition- and time-dependent activity of various TFs as well as during heterodimer formation. We show that a three-layer regulatory cascade whose structure is determined by co-expressed gene modules and their regulators can successfully be modeled using ANNs with a similar configuration.

## Introduction

Gene regulation is a fundamental process for the survival and proliferation of any living cell and is tightly controlled during all cellular states. Cells integrate a wide range of environmental signals in order to regulate their gene expression, which is primarily controlled at the level of transcription initiation. Alterations in the external environment affect the activities of specific proteins known as transcription factors (TFs) which can bind to regulatory regions of their target genes and inhibit or enable their transcription. Gene regulation is a multi-layer hierarchical process (Yu and Gerstein, 2006), where proteins regulate genes that produce other proteins. This interacting machinery comprised of TFs and their target genes can be represented as a directed graph thus forming the transcriptional regulatory network. This is often said to represent the master control system of the cell that orchestrates the differential expression of all genes (Yu et al., 2003; Ihmels et al., 2004) and has received significant attention over the last decade (Ihmels et al., 2004; Tanay et al., 2004; Bruggeman et al., 2008; Petricka and Benfey, 2011).

The organization of regulatory networks reflects the organization of the cellular machinery: it consists of functionally coherent groups, the functional modules (Han, 2008), entities that have been recognized as the basic structural unit in any biological system (Hartwell et al., 1999). In the case of transcriptional regulatory networks, the functional modules are groups of genes that are regulated in a coherent manner and thus behave similarly under similar conditions.

The recent increase in the production and accessibility to large-scale experimental data sets led to the development of computational methods that make use of various algorithmic procedures and integrate heterogeneous biological data aiming to infer functional modules that reflect distinct biological entities (Tanay et al., 2004; Fernández, 2007; Zhao et al., 2007; Bruggeman et al., 2008; Han, 2008). The majority of these methods use expression data from microarray experiments to group genes based on their expression profile, assuming that genes which are co-expressed under certain conditions are likely to be co-regulated and/or belong to the same biological pathway (Segal et al., 2003). Module assignment using gene expression data provides information about the organization of gene activities in different intracellular processes. However, it offers little or no information about the type of regulation (e.g., positive or negative) exerted by various regulators onto the members of a given module under different conditions. This limitation was first confronted by Segal et al. (2003) who applied a probabilistic model on gene expression data to identify the modules, their regulators and the conditions under which this regulation takes place. Since then, numerous studies incorporated heterogeneous experimental data such as motif information (Kundaje et al., 2005; Hu et al., 2008; Lee et al., 2008), chromatin immunoprecipitation (ChiP-chip) (Gao et al., 2004; Imoto et al., 2004; Xu et al., 2004; Youn et al., 2010), and protein interaction data (Tornow and Mewes, 2003; Maraziotis et al., 2008) in order to infer gene functional modules and associated networks.

Tools for inferring such networks include MA-networker (Gao et al., 2004), COGRIM (Chen et al., 2007), ReMoDiscovery (Chen et al., 2007), LeTICE (Youn et al., 2010), and GRAM (Bar-Joseph et al., 2003), among others. These approaches identify direct regulatory interactions but are confined to a single level whereas it has been shown that direct regulators form a bottleneck in the hierarchical structure of the multi-layer regulatory network (Yu et al., 2003). Furthermore, existing network-finding approaches provide mostly qualitative (structural) correlations between regulators and target genes. Attempts to model genetic regulations have been reported, although not very successful (Holter et al., 2000; Liebermeister, 2002; Imoto et al., 2003; Yu et al., 2003; Yu and Li, 2005; He and Zeng, 2006; Pournara and Wernisch, 2007; Wang and Li, 2012). Interestingly, quantitative correlations between expression profiles of TFs and their direct targets were possible when TFs were considered to act in a combinatorial manner, each exerting its regulatory function with a different temporal delay (Boone et al., 2007; He et al., 2007). Finally, networks identified based on expression information, such as frequently done in existing approaches, cannot take into account TFs that are constitutively expressed and yet exhibit a condition-specific regulatory action via the formation of heterodimers (Amoutzias et al., 2008).

In this work, we address the abovementioned limitations by constructing three-layer regulatory cascades having as basis not a single component (gene) but a module of genes along with its regulators, as identified by GRAM (Bar-Joseph et al., 2003), one of the most cited module-finding algorithms (Youn et al., 2010). We then use three-layer Artificial Neural Networks (ANNs) to model transcriptional cascades in order to quantitatively predict the expression profile of any gene in a module, given the expression of its indirect upstream regulators. Our models can be extended to take into account TF dimer formation as well as time-dependent combinatorial regulation that may be exerted by multiple regulators.

Transcriptional regulation is particularly important under conditions influencing the organism's survival, such as severe stress. *Saccharomyces cerevisiae* for example, is an organism whose transcriptional profile under stress has been extensively studied (Bammert and Fostel, 2000; Gasch et al., 2000; Rep et al., 2000; Causton et al., 2001; Kwast et al., 2002), primarily due to the simplicity of its genome and its worth as a biotechnological product (Botstein et al., 1997). Thus, we apply our method to a publicly available dataset concerning the response of *S. cerevisiae* cells to various stress conditions.

## Materials and methods

### Data acquisition and pre-processing

Microarray gene expression data from *S. cerevisiae* cells in response to various environmental stresses (Gasch et al., 2000) were downloaded and used. The dataset comprised of measurements for the expression of 6152 *S. cerevisiae* genes under 19 different conditions over several time points (173 experiments in total) as well as over-expression and knockout experiments. The stress response data included the following conditions: (1) heat shock from 25°C to 37°C, (2) heat shock from various temperatures to 37°C, (3) steady-state temperature growth, (4) temperature shift from 37°C to 25°C, (5) mild heat shock at variable osmolarity, (6) response of mutant cells to heat shock, (7) hydrogen peroxide treatment, (8) response of mutant cells to H_2_O_2_ exposure, (9) menadione exposure, (10) diamide treatment, (11) DTT exposure, (12) hyper-osmotic shock, (13) hypo-osmotic shock, (14) amino acid starvation, (15) nitrogen source depletion, (16) diauxic shift, (17) stationary phase, (18) steady-state growth on alternative carbon sources, (19) steady-state growth at constant temperatures, (20) over-expression studies, (21) knockout experiments for several time points. The dataset included normalized, background-corrected log_2_ Red/Green ratios. Normalization included correction of Cy3 and Cy5 dye biases and background correction to correct for signal intensities outside the spots. The data were log_2_ transformed to avoid fractions in signal ratios. Although normalization represents an important step in microarray data analysis and procedure, in this case, since data were previously normalized, no further normalization was performed. Since many—but not all—experiments were performed in duplicates or triplicates, we used the average expression value in our analysis to ensure equivalent contribution of each data-point in the final analysis and also to avoid having replicates of an experimental condition in both the training and test datasets of the ANNs. Furthermore, in an attempt to focus on responses specific to certain stress conditions, we excluded the over-expression and knockout experiments and divided the rest of the experiments in three main functional categories as shown in Table 1.

**Table 1:** **Stress conditions organized into three categories**.

**Category A (heat shock)**	**Category B (starvation)**	**Category C (oxidative and osmotic shock)**
Heat shock from 25 to 37°C	Amino acid starvation	H_2_O_2_ treatment
Various temperatures to 37°C	Nitrogen source depletion	Menadione exposure
Steady-state temperature	Diauxic shift	Diamide treatment
Heat shock from 37 to 25°C	Stationary phase	DTT exposure
Heat shock at variable osmolarity	Steady-state on alternative carbon sources	Hyper-osmotic shock
Constant temperature growth		Hypo-osmotic shock

Category A includes experiments related to alterations in temperature; Category B includes experiments related to nutrient depletion; and Category C contains experiments related to oxidative and osmotic shock.

A dataset containing genome-wide location analysis for the binding of 106 transcriptional regulators to promoter sequences across the *S. cerevisiae* genome (Lee et al., 2002) was also used in the analysis. In the respective study, the authors used a myc epitope tag for each TFs and performed a genome-wide location analysis using microarrays to detect, through hybridization, those promoter regions of the genome that were enriched in epitope tags after chromatin immuno-precipitation experiments. Binding data are represented as confidence values (p-values) for each microarray spot.

### Identification of gene modules

In order to identify modules of genes that are co-regulated and co-expressed under a set of conditions, we used the GRAM algorithm (Bar-Joseph et al., 2003) (see Box 1 for a brief description). We used the default settings of the algorithm and the pre-processing option (row-wise normalization). Specifically: (1) a binding cutoff *p*-value of 0.001, (2) rejection of a gene if more that 20% of the expression or binding data are missing, and (3) the minimal size of the initial core set of genes forming a module is equal to 3. This first step resulted in a number of gene modules along with their direct regulators which formed the middle-layer of the ANN model.

Box 1. The GRAM algorithm. A heuristic algorithm that identifies modules of co-expressed and co-regulated genes based on expression and DNA-binding data. The algorithm first identifies a core set of genes that are bound by the same set of regulators based on a stringent criterion for the *p*-value binding. For this initial set, the method finds those genes that share a similar expression profile and forms a new core set for which the mean expression profile is calculated. Now an extension step is performed. In this step the algorithm searches for genes that are bound by a more relaxed binding criterion and their expression profile is close to the mean expression profile of the core set of genes. For these genes a combined *p*-value is calculated based on the *p*-values of the regulators for every module. A gene is now added to the initial set if its combined *p*-value is less than a more relaxed binding cutoff.

### Building the regulatory networks

For every GRAM-inferred gene module that is regulated by at least two TFs, we searched for the proteins that regulate these TFs using the YEASTRACT database (www.yeastract.com), to identify the indirect TFs which formed the upper-layer (or input-layer) of the ANN models. Since transcriptional regulation requires the binding of a TF to the promoter region of a gene, in addition to the manually curated bibliographic information, a protein is assumed in this manuscript to transcriptionally regulate the target gene that codes for a specific TF only if it has at least one binding site in the region 1000 base-pairs upstream the promoter of that gene. The region of 1000 base pairs was chosen as this is generally considered the region most likely to contain TF binding sites (Friberg et al., 2005). Binding site data were retrieved from the SGD database (http://www.yeastgenome.org/).

**Figure 1:**
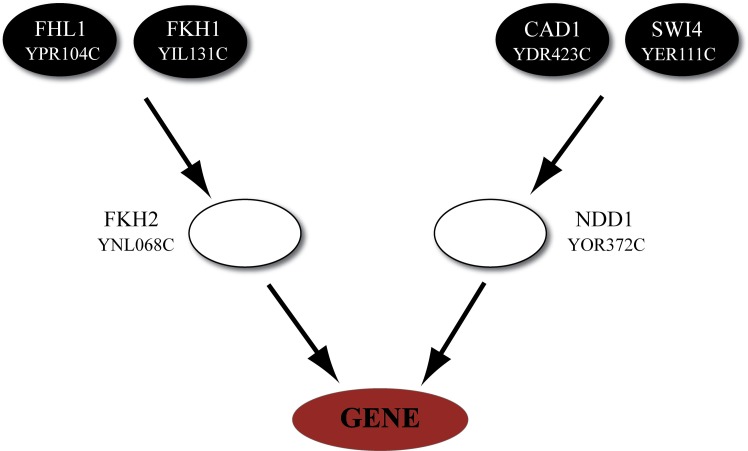
**Representative example of a regulatory cascade**. The regulatory cascade that is formed based on module 20 in Category A (heat shock). The module, as derived from GRAM, consists of seven genes (YLL006W, YDR043C, YML127W, YFL067W, YLL005C, YLL003W, and YJL225C) which are regulated by the transcription factors FKH2 and NDD1. While the biological cascade is common for all genes, a different ANN is trained/validated/tested based on the expression of every target gene in the module. Most of these genes are implicated in the stress response as evidenced from their MIPS annotation (Mewes et al., 1997).

Thus, we built three layer biological cascades (an example is shown in Figure 1) in which the third (output) layer comprises a gene in a module derived by GRAM, the second (middle) layer consists of the TFs that regulate the module and the first (upper) layer includes the TFs that regulate the second layer TFs.

### Modeling and quantitative prediction of gene expression

**Figure 2:**
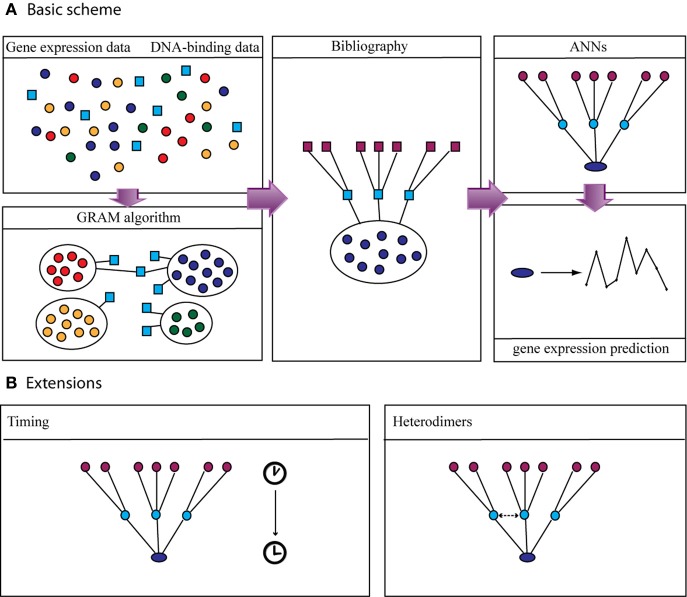
**Flow chart of the proposed method. (A) Left panel:** Gene expression data from microarray studies and DNA-binding data (ChIP-chip) are used as input to the GRAM algorithm in order to identify gene modules and their regulators. **Middle panel:** Bibliographical information from public databases is used to add another layer of regulators and build the three-layer regulatory cascade. **Right panel:** ANNs with the same structure as the biological cascade are built and trained to predict the expression of the target gene given the expression of the upper-layer regulators. **(B)** The basic scheme was extended to include additional biological aspects such as time-delays among the expression of a transcription factor and the expression of the target gene and formation of heterodimers.

Subsequently, we described quantitatively the uni-directional dependencies among the upper-layer and the module output-layer using a well-known machine-learning technique, namely ANNs, which have been previously used to describe interconnections between gene clusters (Huang et al., 2003), identify biomarkers using gene expression data (Pal et al., 2007) and classify gene expression data (Sewak et al., 2009). Unlike previous approaches where the structural characteristics of the ANNs were chosen somehow arbitrarily (Huang et al., 2003), we confined the structure of the ANN to match that of the biological network as this is inferred from expression data, DNA-protein interaction data and bibliographic information (see Figure 2A for an illustration of our methodology).

Each node in the input and middle layers represents one TF and thus the number of nodes in the ANNs equals the number of TFs in the respective biological networks. Examples of such networks are shown in Figures 1, 5, 7, 9 in the Results section. The activation functions in all ANNs are sigmoid-logarithmic in the middle-layer nodes and linear in the output layer. The weights and biases of the network are updated in every training epoch according to the Levenberg–Marquardt optimization (Levenberg, 1944; Marquardt, 1963) implemented in the Neural Network Toolbox in Matlab. For every ANN model the expression profile of the upper-layer TFs is used as input to the model and the expression of the downstream gene is assessed as the output. For each model, training is done using 50% of the experimental conditions and the remaining 50% is used for validation (25%) and testing (25%). Since genes in a module have slightly different expression profiles the procedure of the training/validation/testing is completed independently for every gene. This procedure is repeated 100 times while the training/validation/test data sets are randomly selected for each repetition. The correlation coefficient between the model predictions for the expression of the target gene in the test set and the desired output (the actual expression of the gene) is measured. The model's performance is assessed as the average correlation coefficient taken over the test set for the 100 repetitions and it is considered good if it is higher than 0.70, a threshold which is generally considered stringent in biological simulations (Cohen et al., 2000; Luo et al., 2007).

To assess the statistical significance of the models' prediction accuracy, all ANNs are also trained, validated and tested using randomly shuffled data in the expression profile of the output gene.

### Incorporation of time-delays in TF activities

It is well-known that TFs undergo a number of post-transcriptional and post-translational modifications from the moment they are expressed until they become a functional protein (Benayoun and Veitia, 2009). These modifications are likely to require a significant amount of time causing a “delay” between the expression of a TF and its effect on the expression of a targeted gene. This is supported by previous work, where the expression profiles of certain TFs correlated with the expression profiles of their target genes only when activity delays were considered (Yu et al., 2003; He and Zeng, 2006; Boone et al., 2007; He et al., 2007). Based on the above, the issue of “timing” becomes even more important for indirect regulatory interactions such as those exerted by the upper-layer regulators in our model networks. The time difference between the activity of a given TF and its effect on the expression of a target gene is expected to depend on the environmental stress under study. For example while for a heat shock condition gene expression data are measured over a period of minutes or hours, for nitrogen depletion samples these values are measured over a period of days. Thus, we allowed combinatorial interactions among the time-dependent activities of regulatory TFs that laid two layers upstream in the modeled regulatory cascade and their target genes (see Figure 1—Timing for a graphical illustration). For this reason we use the time-series data of the stress-response dataset and shift the profiles of each TF independently. We aim to identify the combination of the shifted TF activities for which the expression of the target gene can be predicted with the optimum accuracy.

**Figure 3:**
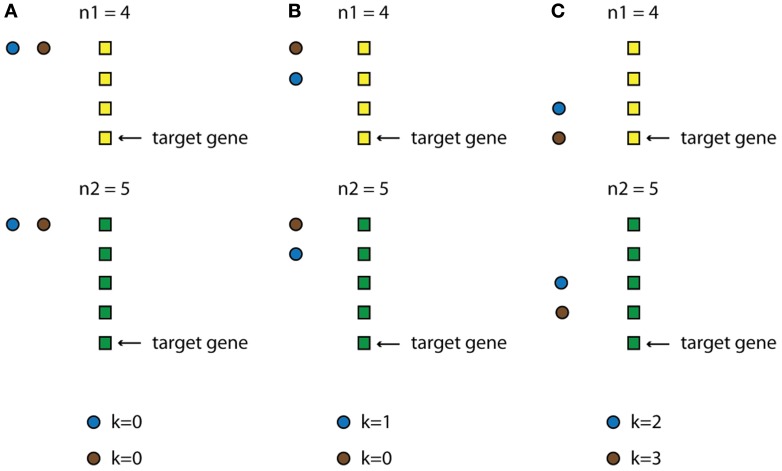
**Schematic example of how the TF-delay table is constructed**. Assume a category that consists of two conditions, with n1 and n2 time points, respectively (black and white rectangles), and a module that, for simplicity, is regulated by two upper-layer TFs (black and gray circles). The condition with the smallest number of time points is n1 so this will be the “leading” condition. Imagine that the target gene is expressed at time point *n*1 = 4 (This ranges from 1 to 4, since the expression profile of the gene is also shifted in time). Every TF is allowed to be expressed at the time points 1, 2, 3, and 4 (*k* represents the steps that each TF is shifted). **(A)** Both TFs are expressed at time point 1 (*k* = 0). **(B)** The first (black) TF is expressed at time point 2 (*k* = 1) while the second (gray) at time point 1 (*k* = 0). **(C)** The first (black) TF is expressed at time point 3 (*k* = 2) while the second (gray) at time point 4 (*k* = 3).

Specifically, we first identify all possible combinations of TF activities when their profiles are allowed to be shifted multiple time steps. The only restriction is that a TF profile cannot be shifted so that the TF appears to be activated after its target gene is expressed. Also, the timing of the expression of every TF cannot exceed the available experimental time points. Thus, the expression profile of every TF i in the data space is shifted *k_i_* time steps backwards, where 0 < *k_i_* < n–1 (n is the total number of time-steps for the condition that has the smallest number of time-steps for this category). As a result, for every set of N TFs we create n·N combinations of possible TF-activity profiles (for an illustrative example see Figure 3 and Box 2). Every such combination is then used as input in the ANNs and the network's performance is assessed as previously discussed.

Box 2. A representative example of the TF-delay implementation. Genes in module 20, Category A (Table 3) are directly regulated by the TFs FKH2 and NDD1 and indirectly regulated by FHL1, FKH1, CAD1 and SWI4. The “Heat shock” category includes the following set of experiments: “Heat shock from 25 to 37°C,” “Various temperatures to 37°C,” “Steady-state temperature,” “Heat shock from 37 to 25°C,” “Heat shock at variable osmolarity,” “Constant temperature growth.” Of the above, sub-categories “Steady-state temperature” and “Various temperatures to 37°C” included only one time point so they were excluded from time-shift analysis. The remaining included 3–9 time points depending on the sub-category. So, the minimum number of available time points is 3, which means that there are two consecutive available steps (this is mentioned as “MAX” in the table) and one non-consecutive. More specifically, the experiment “29C to 33C” includes 3 time points at which the gene expression was measured, namely at 5 min, at 15 min and at 30 min. The available time steps are therefore 5-to-15 min and 15-to-30 min as well as 5-to-30 min. For simplicity we will consider a time delay step of 1 for all TFs. This would mean that either (1) when the expression of the target gene is measured at 15 min, the expression of the TFs is taken to be the one measured at 5 min or (2) when the expression of the target gene is measured at 30 min, the expression of the TFs is taken to be the one measured at 15 min. The same logic applies for time steps larger than one.

### Incorporation of TF dimers

Over 6000 genes in the *S. cerevisiae* genome appear to be regulated by roughly 200 identified TFs (Harbison et al., 2004). While the set of regulators is relatively small, many of them interact with each other in order to form new transcriptional regulators (e.g., TF dimers). As reviewed by Amoutzias et al. (2008), these heterodimers are unstable complexes able to form or decompose according to the required conditions. Moreover, the genes that correspond to each of the components of a heterodimer may have different patterns of expression. It is possible that one of them is constitutively expressed while its partner's expression is altered in a condition-specific manner. Such complex types of regulatory interactions are generally ignored by most module identification methods which focus on the detection of alterations in gene expression in order to classify the genes and their regulators. To address this limitation (Figure 2B—heterodimers), we used protein-protein interaction data (Maslov and Ispolatov, 2007) and searched for possible interacting pairs of the identified TFs in modules with a single regulator. Of the identified pairs we only took into account those for which DNA binding data were available (Lee et al., 2002). Before considering this protein as an additional regulator we test whether it is constitutively expressed in our dataset. This condition is satisfied if at least 95% of all data points across all stress conditions in a certain category do not exhibit more than a twofold change.

## Results

### Identification of transcriptional modules

Application of the GRAM algorithm on DNA binding data as well as gene expression data from *S. cerevisiae* under stress conditions that fall in three categories (1) heat shock, (2) starvation, and (3) oxidative and osmotic stress resulted in the identification of functional modules and their direct regulators. Genes in these modules were grouped according to their expression profiles and the commonality of their regulators. For each category, we identified 87–100 modules each of which is regulated by 1–3 TFs as detailed in Table 2 and in Table S1. The majority of these modules contained a large number of genes that were regulated by a single TF, suggesting a more general role of these modules that spanned over multiple cellular processes. Since our goal was to identify regulatory networks that were specific to stressful stimuli, we limited our analysis to the 69 modules that were regulated by two or more TFs.

**Table 2:** **Information about the modules identified by GRAM**.

	**Category A**	**Category B**	**Category C**
Number of modules	99	100	87
Number of modules with one regulator	73	76	68
Number of modules with >2 regulators	26	24	19
Mean number of genes in modules with one regulator	24.6	22.63	15.45
Mean number of genes in modules with >2 regulators	10.5	11.58	8.47

This table gives information about the number of the genes in the modules as well as the number of their regulators.

### Inference of biological networks

We next searched for the regulators of the TFs that regulate the 69 modules, following the methodology depicted graphically in Figure 2. Based on information from YEASTRACT (www.yeastract.com), for the majority of our modules these regulators were either (1) plentiful (>15), (2) unknown, or (3) did not have a binding site in the promoter region of the gene(s) they regulate (see Methods). As a result, 51/69 modules were excluded from further analysis, leaving a set of 18 modules to be used for building three-layer regulatory cascades. The exclusion of the majority of inferred modules was a result of multiple factors, which, however, did not depend on the 3-layer cascade model itself but on the lack of (unknown TFs or lack of a binding site) or high complexity (too many TFs to find manually) of biological information. If all biological information was available, simulation of all the 3-layer cascades would have been performed. One representative example of such a cascade is shown in Figure 1, which consists of any gene in module 20 (Table S1) whose expression profile is modulated during heat shock (category A), the FKH2 and NDD1 TFs that regulate this module (according to GRAM) and the FHL1, FKH1, CAD1, and SWI4 TFs that regulate FKH2 and NDD1 (according to data from YEASTRACT filtered by our binding criteria). Respective regulatory cascades for all analyzed modules can be found in the Table S2.

### Modeling of biological networks with artificial neural networks

**Figure 4:**
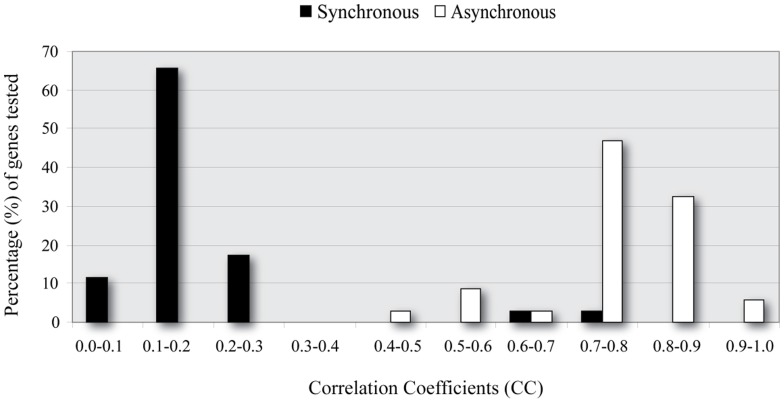
**Correlation coefficient (CC) distributions for Synchronous and Asynchronous ANNs**. ANN models which consider asynchronous interactions (white) achieve significantly higher performance (larger CC values) than synchronous ANNs (black).

Each two layer regulatory cascade was subsequently mapped to a structurally constrained ANN in an effort to quantitatively model the effect of upstream TFs on the expression profile of genes that were two levels downstream in the cascade (see Materials and Methods for details). A total of 70 three layer cascades, corresponding to 70 genes contained in 18 modules identified over all stress conditions were trained, validated and tested. Using as input the expression of the upper level regulators, the performance of each ANN model was evaluated by measuring the correlation coefficient r (CC) between the model output and the true expression profile of the target gene over numerous stress conditions. This approach resulted in a relatively small set (11/70) of ANN models with a CC > 0.7 (see Figure 4 and Table S3). The reasons for this could be numerous, ranging from limitations in the model selection to insufficient biological information. In the following sections we address some of these issues in order to improve the performance of our method.

### Asynchronous networks

One possible limitation of our approach is the assumption that, for a specific condition, the expression of a TF at a given time point has an effect on the expression of its target gene which is evident at the same time point. In other words, the expression profiles of TFs and their targets were assumed to be (relatively) synchronized (Note: Expression values in microarray experiments were measured over distinct time steps in an attempt to capture the gene expression changes during a specific response. However, it is possible that the cause (expression of the TF) and effect (change in the target gene expression) are evident in different sampling times mainly due to the various post-transcriptional modifications that are necessary for the complete activation of the TF). Moreover, in cases where more than one TF regulated a given gene we assume that their effects on gene expression were also synchronous. In reality, this is rarely the case.

To address this problem we next investigated whether a time-shift in the expression profile of each TF, independently of each other, improved the model's performance (see Materials and Methods for details). Specifically, we trained/evaluated and tested the same ANN models using as input the expression profiles of upstream TFs, each of which was shifted one or more time steps backwards in time. Every ANN model implementing a combination of time-shifted TF activities was evaluated using the CC, as previously described. As shown in Figure 4, when a time-shifted (asynchronous) regulation was considered, ANN models could successfully predict the expression profiles of target genes in the majority of the simulated regulatory cascades. It is important to note that usually more than one combinations lead to accurate models (i.e., CC > 0.7). This is rather intuitive since transcriptional regulation is critical for the cell's survival and it is often achieved via alternative pathways (Wagner and Wright, 2007).

A representative example of the improvement in ANN performance via the incorporation of TF delays is shown in Table 3. The table lists the correlation coefficient of the ANN models for three target genes in the network of Figure 1, namely genes YDR043W, YML127W, and YJL225C. “Synchronous” indicates the prediction accuracy of the ANN model when no time-shifts in the TF activities were considered while “Asynchronous” corresponds to the ANN model that incorporates the optimal combination of TF delays, namely the one leading to the highest CC. For gene YDR043W for example, the effects of the TFs FHL1, FKH1, CAD1, and SWI4 were evident in the expression of the target gene after 0, 2, 0, and 1 time steps, respectively. The same TFs appeared to regulate the other two genes in this module with different time delays, indicating that the effect of the same TF under the same conditions on the expression of its target was gene-specific.

**Table 3:** **Maximum correlation coefficients for three cascades modeled by synchronous or asynchronous ANNs**.

**Module 20**	**Correlation coefficient**		**Transcription factor delays**				
	**Synchronous**	**Asynchronous**	**FHL1**	**FKH1**	**CAD1**	**SWI4**	**MAX**
YDR043W	0.65 ± 0.11	0.86 ± 0.13	0	2	0	1	2
YML127W	0.73 ± 0.11	0.85 ± 0.11	0	2	1	1	2
YJL225C	0.48 ± 0.15	0.72 ± 0.14	0	0	0	1	2

In all cases, asynchronous ANNs achieve higher performance. The number of possible time-shifts for each TF depends on the number of experimental time-points available for each condition. This number is indicated with MAX while the number of time points a transcription factor is expressed before the expression of the target gene is referred to as transcription factor delays.

Detailed information regarding the optimal time-shifted combinations of TFs for all simulated cascades can be found in Table S4. It is striking that in the majority (30/34) of simulated regulatory cascades there is a significant improvement in the prediction accuracy of the ANN models, suggesting that the transcriptional regulation of most downstream genes is likely to result from of a time-dependent combinatorial activation of upstream TFs.

### Incorporation of TF-dimers

An important limitation of the GRAM algorithm that may have influenced the ANN models is the assumption that TFs regulating a given gene should be differentially expressed under the various stress conditions. This assumption is frequently violated in cases where two TFs form a dimer in order to exert their regulatory action. To address this issue, we also considered constitutively expressed proteins that are found to interact with already identified regulators. Two such proteins were found in our dataset, namely PHO2 and HIR1, which are known to interact with BAS1 and HIR2, respectively.

**Figure 5:**
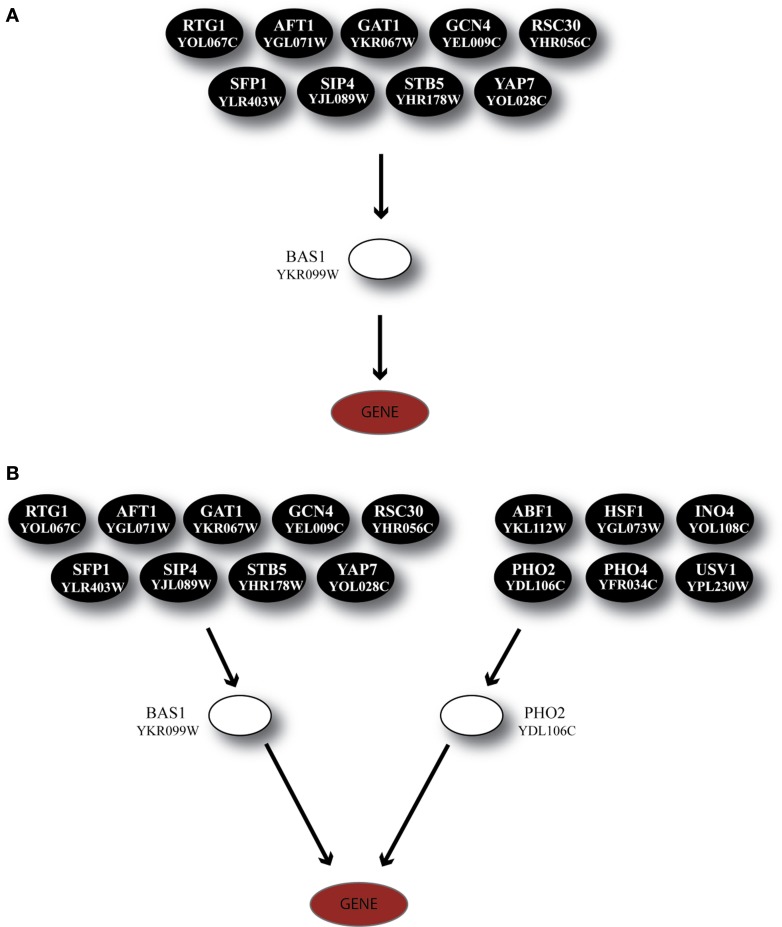
**Regulatory cascades containing the TF BAS1 as the GRAM-identified regulator. (A)** Regulatory cascade where only BAS1 is considered as a regulator. **(B)** Regulatory cascade where BAS1 as well as PHO2 are considered as regulators of the target gene. These cascades are found in all three stress categories but correspond to different gene modules as identified by the GRAM algorithm.

**Figure 6:**
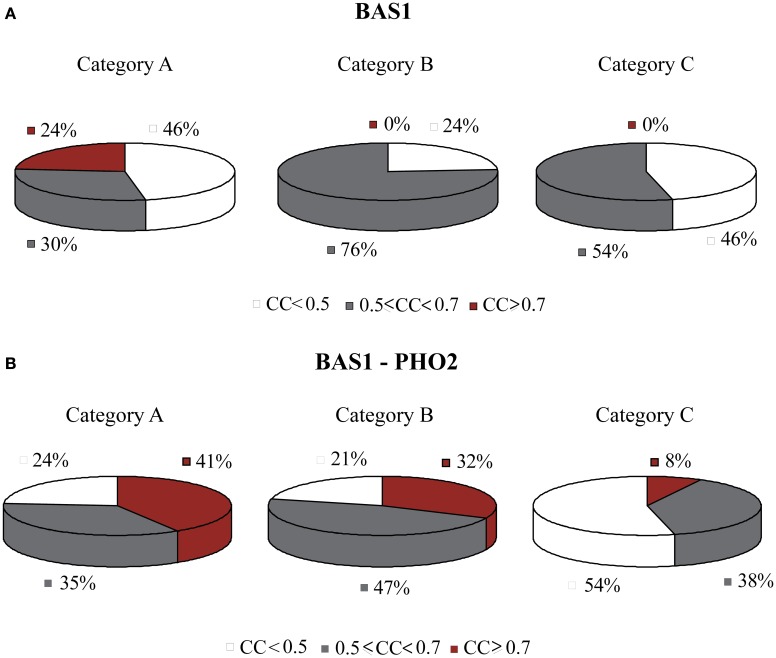
**Pie charts showing the grouped correlation coefficients (CC) of the ANN models in the three stress categories. (A)** Results correspond to genes in modules regulated by BAS1 alone. Successful ANNs (CC > 0.7) are found only for stress conditions in Category A (heat shock). **(B)** Results correspond to genes in modules regulated by the BAS1-PHO2 dimer. In this case, there is a significant increase in the correlation coefficient of ANNs over all three stress categories.

The TF BAS1 has been implicated in the biosynthesis of histidine, purine and pyrimidine pathways (Tice-Baldwin et al., 1989; Daignan-Fornier and Fink, 1992). According to GRAM, it appeared as a unique regulator for 17 genes in Category A, 21 genes in Category B, and 13 genes in Category C. Moreover, BAS1 is itself regulated by 9 TFs, according to the YEASTRACT database. ANN simulation of the respective regulatory cascade (shown in Figure 5A) showed that in Category A (heat shock) the expression profiles of only 24% of the genes can be predicted with a correlation coefficient above 0.7 while for the rest of the genes in this category as well as genes in the other two categories prediction accuracy was much lower (Figure 6A).

Based on experimental data for protein-protein interactions, PHO2 interacts with BAS1 and is likely to act as a co-regulator as its presence is necessary in order for BAS1 to exert its regulatory action (Maslov and Ispolatov, 2007). In addition, PHO2 was constitutively expressed under both normal and stress conditions in our dataset. Incorporation of both TFs in the ANN model (cascade is illustrated in Figure 5B) significantly improved the model performance, particularly for genes in Categories A and B and less in Category C (see pie charts in Figure 6B). Specifically in Category A, 41% of gene profiles could be predicted with high accuracy, while this number dropped to 32% and 8% for Categories B and C, respectively. Among the ANNs with improved performance, the model corresponding to YOR224C (RPB8) appeared in all three categories. RPB8 is part of the RNA-polymerase III subunit (Archambault and Friesen, 1993; Chédin et al., 1998; Geiduschek and Kassavetis, 2001) and at least three other genes in the same module encode proteins related to ribosomal components. There is supporting evidence for the involvement of BAS1 and RNA-polymerase and ribosomal proteins in other common regulatory pathways (Som et al., 2005; Kresnowati et al., 2006), an indication that BAS1 is indeed a necessary regulator in various aspects of the cellular machinery.

**Figure 7:**
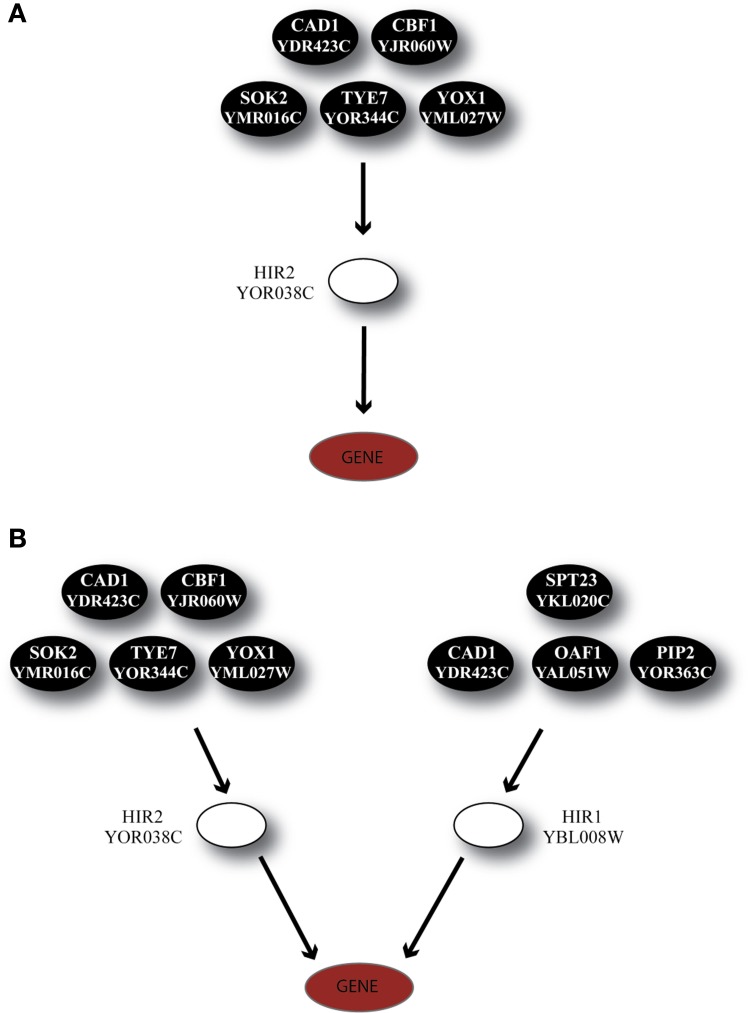
**Regulatory cascades containing the TF HIR2 as the GRAM-identified regulator. (A)** Regulatory cascade where only HIR2 is considered as regulator. **(B)** Regulatory cascade where HIR2 and HIR1 are considered as regulators of the target gene.

**Figure 8:**
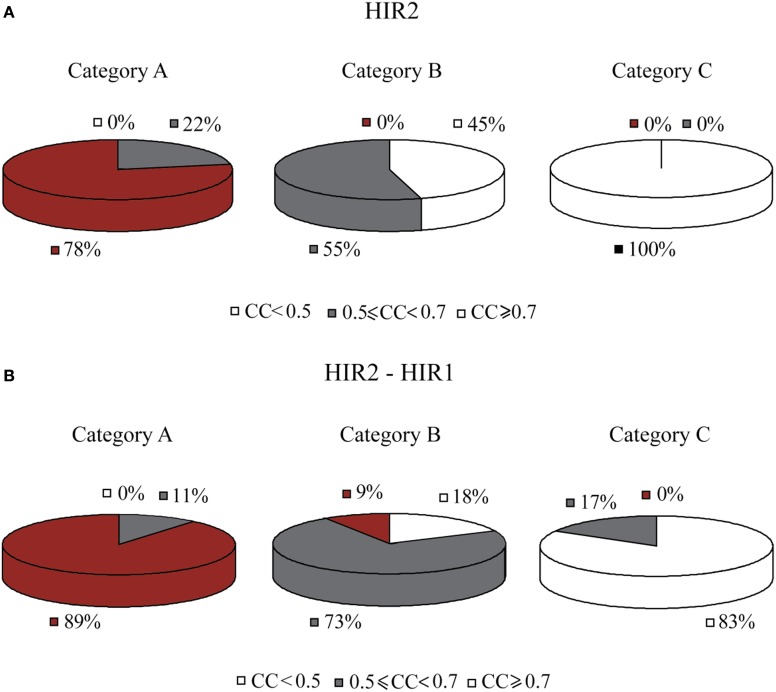
**Pie charts that show the grouped correlation coefficients (CC) of the ANN models in the three stress categories. (A)** Results correspond to genes in modules regulated by HIR2 alone. ANNs with CC > 0.7 are only seen in Category A (heat shock). For Category C in particular, the correlation coefficient for all genes is very low (CC < 0.5). **(B)** Results correspond to genes in modules regulated by HIR2 and HIR1. In this case the ANN performance is slightly improved in all three categories.

Another regulator, HIR2, was also identified by GRAM as a unique regulator for 19 genes in Category A, 21 genes in Category B and 5 genes in Category C. HIR2 was itself regulated by 5 TFs as illustrated in Figure 7A. The ANN models that simulated this cascade achieved high performance for 78% of the target genes in Category A, but poor performance for the genes in the remaining Categories (pie charts in Figure 8A).

According to experimental evidence, HIR2 acted as a co-regulator of HIR1 for the transcription of histone genes (Sherwood et al., 1993; Desilva et al., 1998). Given that HIR1 was constitutively expressed in over 95% of our stress conditions and interacted with HIR2, a biological cascade that involved both TFs as middle-layer regulators was built (Figure 7B). Simulation of this cascade with ANNs resulted in high performance (CC > 0.7) for 89% (17/19) of the genes in Category A. A large fraction (6/19) of the genes in this category is of unknown function, while 4/19 genes have been associated with the ribosomal machinery and the rest share various other functions. Performance in Category B also improved with 9% of the ANNs achieving a CC above 0.7 while in Category C, although the performance of all ANNs was improved, no gene could be predicted with a CC > 0.7 (Figure 8B). The dramatic difference between ANN model performance in the three categories could suggest that the formation of the heterodimer HIR2-HIR1 is condition-dependent, as is the case for the majority of the known heterodimers (Amoutzias et al., 2008).

**Figure 9:**
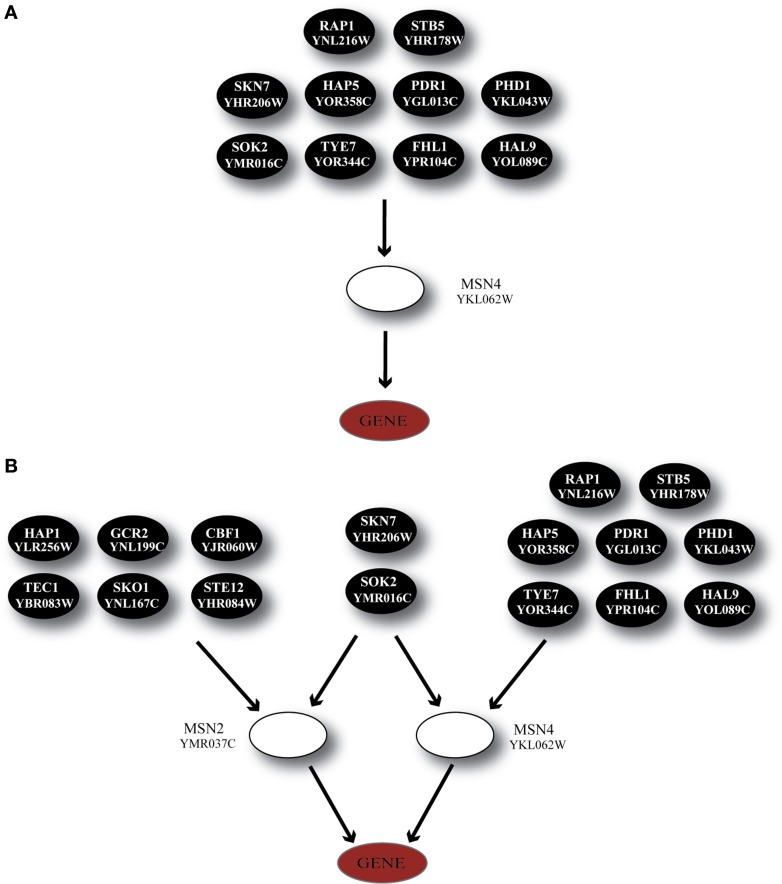
**Regulatory cascades containing the TF MSN4 as the GRAM-identified regulator. (A)** Regulatory cascade where only MSN4 is considered as a regulator. **(B)** Regulatory cascade where MSN4 and MSN2 are considered as regulators of the target gene.

Finally, a special case of transcriptional regulators that are known to have a key role in the stress response in *S. cerevisiae*, MSN2 and MSN4 (Martinez-Pastor et al., 1996; Gasch and Werner-Washburne, 2002), were included in our analysis. In accordance to experimental findings (Haitani and Takagi, 2008), MSN2 was constitutively expressed across all stress conditions in our dataset; subsequently, the GRAM algorithm identified only MSN4 as a transcriptional regulator for 24 genes in Category A, 24 genes in Category B and 16 genes in Category C (the regulatory cascade that includes MSN4 as a middle-layer regulator can be seen in Figure 9A).

**Figure 10:**
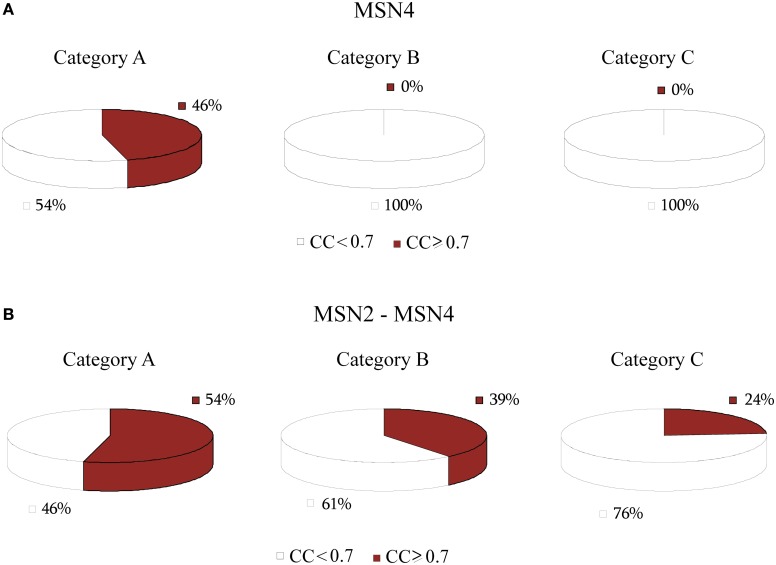
**Pie charts that show the grouped correlation coefficients (CC) of the ANN models in the three stress categories. (A)** Results correspond to genes in modules regulated by MSN4 alone. ANNs with CC > 0.7 are only seen in Category A (heat shock) whereas no good predictions were found in categories B and C. **(B)** Results that correspond to genes in modules regulated by MSN4 and MSN2. In this case the ANN performance is improved in all three categories, with Category A having the majority of the successful ANNs, in agreement with experimental evidence.

Considering the importance of both regulators and their ubiquitous presence in stress response we built the regulatory cascade illustrated in Figure 9B which includes both MSN2 and MSN4 as well as their direct regulators. Figure 10 illustrates the performance of the ANNs in the three categories, grouped as models with CC that exceeds the 0.7 threshold and those for which the CC < 0.7 for MSN4 alone (Figure 10A) and MSN4 and MSN2 cascades (Figure 10B). ANN models that contained only MSN4 as a middle-layer regulator had a relatively good performance in Category A and a poor performance in the remaining categories. ANNs that consider both MSN4 and MSN2 had a good performance in all categories. Specifically, in category A (heat shock) more than half of the gene expression profiles could be predicted with high accuracy and it is important to note that the average CC of these ANNs was 0.85. The very high performance under heat shock (in both MSN4 alone and MSN2/4 models) could be related to the unique role of the MSN2/4 TFs to stress responses associated with alterations in temperature. Experimental evidence has shown that despite their overall function as stress response factors, they seem to be especially implicated in pathways that are triggered during heat shock (Ernst et al., 2007).

To ensure that improved ANN performance was not simply due to the addition of extra free parameters (namely the extra weights between the second middle layer node and its upper layer regulators as well as the weight between the output gene and the added middle layer node), we replaced the PHO2 and HIR1 TFs, respectively, with five (each) randomly selected TFs. All of the randomly selected replacements were regulated by the same number of upper layer TFs as the replaced factor to ensure the same degree of flexibility (same number of free parameters/weights) in the resulting ANN models. Specifically, the following pairs of second layer TFs were used: (a) BAS1-YBR137W, (b) BAS1-SLY1, (c) BAS1-SAT4, (d) BAS1-CCS1, and (e) BAS1-THR1 instead of the original BAS1-PHO2 dimer and (a) HIR2-YDR249C, (b) HIR2-PMT1, (c) HIR2-YDL026W, (d) HIR2-YJR085C, and (e) HIR2-HIS6 instead of the HIR2-HIR1 dimer. This procedure resulted in ten additional three-layer cascades and a total of 10 × 86 (10 × 41 for each BAS1-pair and 10 × 45 for each HIR2-pair, see previous paragraphs) new ANN models. In all cases the ANN models resulting from the randomly selected TF pairs had poorer performance (fewer ANN models with CC > 0.7) than the respective ANNs corresponding to the original dimers. Moreover, in several cases performance was worse than the ANNs incorporating only one of the TFs (as opposed to the dimer), indicating that improved performance does not result from the added flexibility provided by the extra free parameters.

### Statistical assessment of ANNs' performance

**Figure 11:**
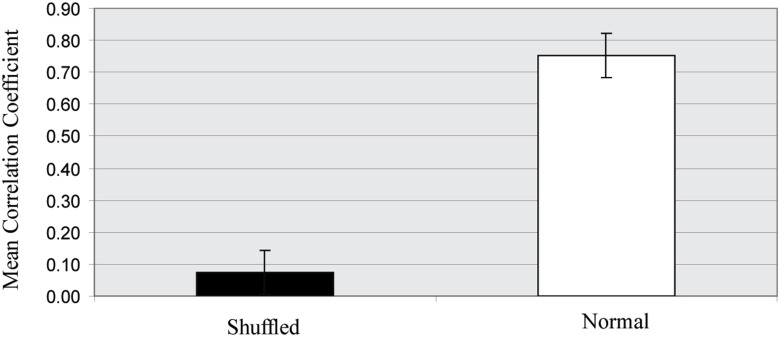
**Correlation coefficients of the normal and shuffled data**. Bars show the mean and standard deviation of the correlation coefficients achieved by all ANN models when either the normal (white) or randomly shuffled (black) expression profiles for the target gene where used to train the models.

To assess the accuracy of our ANN models in predicting the expression profile of downstream genes we shuffled the expression values of each target gene and re-trained the models. We found that for all ANNs with CC > 0.7, the correlation coefficient for the shuffled data dropped dramatically, indicating that the performance of our ANN models is far from random chance (see Figure 11 for a detailed comparison). For the asynchronous networks in particular, the ANN models corresponding to the various combinations of time-shifted TF activities were also simulated using 100 trials where the expression profile of the target gene was randomly shuffled and the resulting CC was recorded. In all cases, the CC of the optimal set of time-shifted TF activities was found to be significantly higher than the maximum CC obtained by the shuffled ANNs, indicating that the improved performance in asynchronous networks was not a result of overfitting.

## Discussion

In this work we introduced a semi-dynamic method for modeling the structure of three-layer transcriptional regulatory cascades based on ANNs. The method calculates quantitatively the expression profiles of *S. cerevisiae* genes during various stress conditions, based on the expression of their upstream regulators. *S. cerevisiae* was selected because it is a well-studied organism with an important biotechnological role (Botstein et al., 1997). The availability of a plethora of expression studies concerning the response of *S. cerevisiae* to different environmental changes (Bammert and Fostel, 2000; Gasch et al., 2000; Rep et al., 2000; Causton et al., 2001; Kwast et al., 2002) underlies the importance of this mechanism and provides the large amount of data required for a quantitative approach like the one proposed here.

Our approach depends on the use of publicly available software tools (e.g., GRAM or any other module finding tool) for the identification of groups of genes which share common expression profile and a common set of regulators. Using these modules, along with information regarding protein-DNA and protein-protein interaction data, we extend the regulatory modules into three-layer cascades by adding another level of regulation. These cascades are then simulated using ANNs where the expression of the upper level regulators is used to predict the expression of the downstream target gene(s).

Initial use of the ANNs under the assumption that expression profiles corresponding to regulators and regulated genes evolve in synchrony over time and under various conditions, resulted in a relatively poor outcome: only 11 out of 70 models had a high performance (CC > 0.7). Incorporation of time-delays between the profiles of regulators and those of regulated genes, which can vary independently for each regulator, resulted in a massive increase in the percentage of successful ANN models:

Thirty out of thirty four networks tested had a significant improvement in their performance, validating the intuitive assumption that a TF can be expressed several time steps before its effect is evident in the expression of the target gene and that regulators that target the same gene may exhibit their activity in a combinatorial manner. Identification of the optimal combination of TF activities for which the expression of a downstream target gene can be predicted with high accuracy leads to a working hypothesis that describes not only the regulatory components for this gene (network structure) but also their interaction over time (network functionality over time). An important finding is that for every regulatory cascade there is more than one optimal combination of transcriptional activities, in agreement with the observed flexibility of biological systems to overcome perturbations that could hinder their regulatory program.

The method was also found to perform well in three cases where known regulators, which are constitutively expressed under stress conditions, were introduced in the middle layer. Incorporation of protein interaction data as an indication of heterodimer formation to previously identified cascades resulted in a significant improvement in the performance of ANN models. Specifically, we incorporated the interaction of BAS1 with PHO2 and the interaction of HIR2 with HIR1. In the latter case, model performance was condition-dependent with heat shock and starvation categories having the highest performing models, thus pinpointing to the specific stress conditions that favor the formation of the given heterodimer.

Existing network inference approaches range in the field between two extremes. Considering the gene as only existing in two discrete situations that is on and off, as in Boolean approaches (Bornholdt, 2008) or offering an analytical description of the gene state using differential equations (Sakamoto and Iba, 2001). The main limitations of these approaches is the assumption of binary values for the expression of a gene and the requirement for a large amount of parameter values that are not usually available and therefore the restriction of the method to a very small portion of the regulatory network, respectively. Bayesian networks (BN) (Hecker et al., 2009) represent the regulatory interactions by probability in a predefined graph and they are based on identifying the conditional probabilities that best match the expression data on this graph. Their main advantages include the offering of a flexible framework for the combination of different types of data and the ability to avoid over-fitting a model to the training data.

Our method covers these advantages and overcomes the bottleneck of the graph identification by restricting the model structure through the application of clustering methods and bibliography. We demonstrate that these models can capture simple as well as more complicated aspects of biological regulation ranging from semi-dynamic predictions of gene expression profiles over multiple conditions to incorporation of additional types of regulation. Although ANNs have been used in the past to model simple regulatory cascades with moderate accuracy, they did not reproduce the structure of biological networks which enforces several constrains and did not extend to more than one level of regulation (Hart et al., 2006). Our findings show that ANNs can be used to simulate indirect, time-dependent interactions between transcriptional regulators and genes downstream in a cascade as well as their evolution over multiple conditions and time points.

In summary, we propose a quantitative method for modeling biological cascades based on the ANN formalism. Compared to existing methods for network inference and parameter optimization, the proposed scheme uses a combination of a data driven process that identifies clusters of co-expressed and/or co-regulated genes along with a knowledge—driven feature selection approach of previously identified protein-protein interactions in order to reduce the dimensionality of the network components.

Our method offers the algorithmic simplicity of the Boolean network approach using real expression data and although the simulation of these data lacks the analytical description offered by differential equation methods, it can be applied in three layer regulatory networks. Albeit our method was implemented using *S. cerevisiae* data, it is readily applicable to any other organism and/or condition influencing gene transcription. While our method is restricted to time-course gene expression data and requires previous knowledge on gene regulatory interactions, it can be used to predict the expression of downstream regulated genes based on the expression of their regulators and can predict the expression in time points that are not present in the experimental dataset. Moreover, it could be used to identify the time dependencies of regulatory interactions and identify the optimal timing of regulatory combinations. Finally, using our method one could propose candidate co-regulatory pairs of TFs (over a pool of available TFs) based on the increased performance of the ANN for each pair as well as the condition under which this pair is formed. Future work can address the limitations of our approach regarding the identification of upstream regulators, by incorporating an automated search method for inferring these molecules and their interactions from public databases.

## Author contributions

Maria E. Manioudaki developed the methods, analyzed, and interpreted the data and wrote the paper. Panayiota Poirazi designed the study, wrote the paper and supervised the project.

### Conflict of interest statement

The authors declare that the research was conducted in the absence of any commercial or financial relationships that could be construed as a potential conflict of interest.

## Supplementary material

Table S1 **Functional modules**. This table shows the genes in every module identified by GRAM, grouped according to similarity in their expression profiles.

Table S2 **Transcriptional regulatory networks**. The genes in every module are regulated by the 1st-layer regulators. Each 1st-layer regulator is regulated by the 2nd-layer regulators that lie in the box on the right.

Table S3 **Network performance**. Average correlation coefficients and their standard deviation for Synchronous and Asynchronous networks. The correlation coefficients of corresponding shuffled networks along with the *p*-values for the highest CC among Synchronous and Asynchronous networks is also shown.

Table S4 **TF delays**. Tables showing the steps of each TF delay in the networks. MAX shows the maximum number of time-delays available for the particular network.
